# Priming healthy eating. You can't prime all the people all of the time^☆^

**DOI:** 10.1016/j.appet.2015.01.018

**Published:** 2015-06-01

**Authors:** Suzanna E. Forwood, Amy L. Ahern, Gareth J. Hollands, Yin-Lam Ng, Theresa M. Marteau

**Affiliations:** a Behaviour and Health Research Unit, Institute of Public Health, University of Cambridge, Cambridge, United Kingdom; b Medical Research Council Human Nutrition Research, Cambridge, United Kingdom

**Keywords:** Priming, Food choice, Advertisements, Healthy eating

## Abstract

In the context of a food purchasing environment filled with advertising and promotions, and an increased desire from policy makers to guide individuals toward choosing healthier foods, this study tests whether priming methods that use healthy food adverts to increase preference for healthier food generalize to a representative population. *Methods*In two studies (Study 1 n = 143; Study 2 n = 764), participants were randomly allocated to a prime condition, where they viewed fruit and vegetable advertisements, or a control condition, with no advertisements. A subsequent forced choice task assessed preference between fruits and other sweet snacks. Additional measures included current hunger and thirst, dietary restraint, age, gender, education and self-reported weight and height. *Results*In Study 1, hunger reduced preferences for fruits (OR (95% CI) = 0.38 (0.26–0.56), *p *<* *0.0001), an effect countered by the prime (OR (95% CI) = 2.29 (1.33–3.96), *p* = 0.003). In Study 2, the effect of the prime did not generalize to a representative population. More educated participants, as used in Study 1, chose more fruit when hungry and primed (OR (95% CI) = 1.42 (1.13–1.79), *p* = 0.003), while less educated participants' fruit choice was unaffected by hunger or the prime. *Conclusion*This study provides preliminary evidence that the effects of adverts on healthy eating choices depend on key individual traits (education level) and states (hunger), do not generalize to a broader population and have the potential to increase health inequalities arising from food choice.

## Highlights

• Mock adverts were used to prime healthy eating in two studies.
• The primes increased fruit preference in more educated and hungry individuals.
• Priming did not alter fruit preference in less educated or non-hungry individuals.
• The mechanism of prime action may be dependent on participant characteristics.
• Primes effective in some participants cannot be assumed to be effective in others.

## Introduction

Cues in our environment alter what we eat (e.g. Bell, Meiselman, Pierson, & Reeve, 1994; Jacob, Gueguen, & Boulbry, 2010) and the amount we eat (e.g. Harris, Bargh, & Brownell, 2009). More generally, the omnipresent imagery of food in the modern built environment has been argued as contributing to rising rates of obesity (Cohen, 2008), with adverts for unhealthier foods identified as a significant driver of increased unhealthy eating behavior (Mills, Tanner, & Adams, 2013). There is now interest from policy makers in determining whether similar interventions can be used to shift food selections toward healthier foods (Marteau, Hollands, & Fletcher, 2012), raising the question of whether healthy eating can be primed by incidental cues.

Priming is a psychological effect in which exposure to a stimulus is found to increase the accessibility of semantically related concepts, reflected in faster reaction times (Neely, 1977) or in recognition of more degraded images (Gollin, 1960). Recent work in social psychology has explored priming of non-laboratory behavior. For instance, activation of a trait construct or a stereotype in one context (such as the construct of intelligence) results in modification of an unrelated behavior to be consistent with that construct (such as higher test scores) without raising conscious awareness of a link between the two (Bargh, Chen, & Burrows, 1996; Bargh & Ferguson, 2000; Dijksterhuis & van Knippenberg, 1998). A distinct but related effect is goal priming, in which a goal, the representation of a desired end-state, is activated and results in behavior consistent with goal attainment. Goal priming can contrast with other forms of priming in a number of ways (Forster, Liberman, & Friedman, 2007), for instance, priming of one goal might inhibit activation of a competing goal (Stroebe, Mensink, Aarts, Schut, & Kruglanski, 2008).

The use of priming to guide people toward making healthier food choices with primes that are feasible for use in real food choice environments has a modest but growing literature associated with it (Table 1). While these studies are quite heterogeneous in their design, they do suggest some potential in using primes to improve the healthiness of food choices, predominantly by reducing the rate of purchase and consumption of high fat and sugar snack foods. However, published studies do not yet tell us whether such interventions could be effective across a population and in particular among those who are more socially deprived and who purchase a poorer quality diet (Pechey et al., 2013).

It is notable that in many of the existing studies the priming effect is selectively observed in a subset of individuals: those with a food related trait of being either overweight (Papies, Potjes, Keesman, Schwinghammer, & van Koningsbruggen, 2013) or a restrained eater (Buckland, Finlayson, & Hetherington, 2013; Coelho, Polivy, Herman, & Pliner, 2009; Papies & Hamstra, 2010; van Koningsbruggen, Stroebe, & Aarts, 2011). The remaining studies don't report interactions between restraint status and the effect of the prime, or do not report dietary restraint (Boland, Connell, & Vallen, 2013; Gaillet, Sulmont-Rosse, Issanchou, Chabanet, & Chambaron, 2013; Gaillet-Torrent, Sulmont-Rosse, Issanchou, Chabanet, & Chambaron, 2014). These data suggest that the presence of primes to improve the health profile of food choices in a real food retail environment will be particularly effective for those with higher levels of dietary restraint. But since individuals who stand to gain from such an intervention, such as those with a high body mass index (BMI) or a lower socio-economic status, show the same levels of dietary restraint as the remainder of the population (Dykes, Brunner, Martikainen, & Wardle, 2004; Johnson, Pratt, & Wardle, 2012), such selectively effective interventions will not necessarily benefit those who need it most.

Additionally, papers seldom report sufficient demographic detail to determine the extent to which study participants are representative of the wider population, and the recruitment procedure described suggest the use of a convenience sample rather than a representative sample. Three studies recruited participants from within their university (Boland et al., 2013; Buckland et al., 2013; Coelho et al., 2009), likely biasing recruitment in favor of 18 to 25 year-olds with post-18 education to a level that is not seen in the wider population, and only one of the priming studies (Papies et al., 2013) reported a measure of socio-economic status.

In an effort to identify a prime to influence grocery shopping in a general population, our study sought to assess the effectiveness of a prime for healthy eating that was designed to increase preference for fruit over high fat and sugar snacks without being contingent on restraint status (Study 1). Alongside this outcome measure, participants were asked to make size estimates of foods which were congruent and incongruent with a healthy eating goal as increased size estimation of goal-congruent items has been used as a measure for assessing goal activation (Bruner, 1957; Veltkamp, Aarts, & Custers, 2008b; see Measures). This prime was then tested within a sample representative of the population (Study 2).

## Study 1

This study used two prime components designed to prime a healthy eating goal by presenting healthy eating (specifically fruit and vegetable consumption) in a positive manner not contingent upon restraint or dieting status. Three adverts were designed to enhance the motivational value of fruits and vegetables by pairing their consumption with positive affect (Veltkamp et al., 2008b) and highlighting it as a social norm (Thaler & Sunstein, 2008). Similarly, a banner containing images of fruit and vegetables alongside wording indicative of positive mood was presented above a questionnaire.

Participants were told that they were being asked to complete a number of unrelated tasks on the topic of food. This served to reduce the likelihood that participants would be aware of a link between the prime adverts and the food preference outcome, therefore reducing demand effects on the primary outcome. For the same reason, participants received an additional task (the questionnaire below the banner image) asking them to report their food shopping habits.

Work carried out by Veltkamp et al. (2008b) indicates that primes only alter behavior when in a state of raised motivation, such as when hungry; therefore, in our study we did not predict a main effect of prime on the outcome measure. Instead, we predicted that the effect of prime would be moderated by self-reported hunger. A second factor, cognitive load, was also hypothesized to interact with the prime in influencing food preference. Loading cognitive capacity, e.g. by requiring a string of numbers to be held in working memory, has been shown to cause pre-existing latent or implicit goals to drive behavior (Shiv & Fedorikhin, 1999), with these goals driving behavior through their affective value rather than a cognitive value judgment. Loading cognitive capacity therefore has the potential to facilitate the expression of a prime that links healthier eating with positive mood, such as the one used in the current study.

In order to capture the generalized effect of the prime on a goal of eating the broader category of fruits and vegetables, rather than on the goal of eating the particular foods in the prime material, the outcome measures assessed increased preference for fruits (fresh pineapple, apple, peaches, melon slices, cherries, strawberries, dried apricots) that did not feature in the prime (banana, orange juice, vegetable soup). In addition, fruits made a more plausible alternative to sweet snacks in the food preference task than vegetables.

We tested two hypotheses:

(1) The prime activates the goal of eating fruits and vegetables in participants with high hunger, as measured by (a) an increased size estimation of a fruit, and (b) an increased frequency of fruit selection in a food preference task.
(2) The prime activates the goal of eating fruits and vegetables under cognitive load, as measured by (a) an increased size estimation of a fruit, and (b) an increased frequency of fruit selection in a food preference task.

## Method

### Design

Study 1 used a 2 (prime type) by 2 (cognitive capacity) between subjects design. The prime factor comprised a prime condition (with adverts and healthy eating banner) and a control (no adverts and non-food banner). The cognitive capacity factor included a cognitive load condition and a control condition*.*

### Participants

The participants used in this study comprised 143 men and women who were registered with a local volunteer research panel (http://www.cambridgebioresource.org.uk/) and had no recorded health problems (mean (SD) age: 43.6 (14.96) years; BMI: 25.3 (6.9); 68.5% female, see Supplementary Table S1). Participants were compensated for taking part with a £3 shopping voucher. This research was approved by the University of Cambridge Psychology Research Ethics Committee (PRE 2011-57).

### Interventions

The priming condition intervention consisted of an advert rating task and a manipulation of a banner above the food shopping questionnaire. For the advert rating task, mock fruit and vegetable adverts were created using photographic images of people consuming a banana, orange juice, and vegetable soup, each paired with wording that indicated positive affect (e.g. “Give us a smile”) or a social norm (e.g. “everyone's favorite”) (Supplementary Fig. S1). The advertisement designs were informed by a marketing consultant with extensive industry experience of food retail, in addition to feedback from pilot studies with volunteers. Participants were asked to rate each advert using a seven-point scale (ranging from 1 “not at all” to 7 “very”) on six attributes: effective, fun, off-putting, memorable, uninformative, and plausible. This task was included to support the assertion of completing multiple tasks, and these data will not be reported further. The control group did not complete an advert rating task.

The food shopping habits questionnaire asked participants about grocery shopping responsibility, frequency and retail outlets used (Food Standards Agency, 2011). This supported the assertion of completing unrelated tasks, and also acted as a task to complete with the presentation of a banner at the top of the screen. In the prime conditions this banner featured an image of a wide selection of fruits and vegetables, and in the no-prime conditions the banner featured an abstract splash of colored paint (these being matched for size and color characteristics). Both banners included the tag-line “Feel Great”.

Participants in the cognitive load conditions were given a six letter unpronounceable consonant string and asked to retain this in their memory while performing the size estimation task, and another consonant string to retain in their memory while performing the food preference task (Van Boven & Robinson, 2012). Participants in the no-load condition were not asked to complete this task.

### Measures

#### Size estimation

Participants were asked to estimate (in either centimeters or inches) the size of three items: an apple (a means to achieving a healthy eating goal); a chocolate muffin (a means to achieving an indulgent eating goal); and a book (control item) (Bruner, 1957; Veltkamp et al., 2008b). All responses were converted into centimeters for analysis.

#### Food preference

Participants were presented with seven pairs of snacks; one of the items in the pair consisted of fruit i.e. fresh pineapple, apple, peaches, melon slices, cherries, strawberries, dried apricots, while the other item consisted of a sweet dairy or pastry snack, i.e. iced doughnuts, blueberry muffins, iced bakewell tarts, a slice of apple pie, chocolate profiteroles, ice-cream scoops in assorted flavors, chocolate-chip cookies. Participants were asked for each pair presented “Which food would you prefer to have right now?” (Finlayson, King, & Blundell, 2007; Shiv & Fedorikhin, 1999). The outcome measure was the total number of fruits chosen (taking values from 0 to 7).

#### Hunger and thirst

Prior to viewing the prime, or any other material, participants rated their hunger and thirst levels each on a seven point scale (anchored from 1 “not at all” to 7 “very”). Thirst was included to support the assertion of completing unrelated tasks.

#### Dietary restraint

Subsequent to testing, participants completed the Restraint Scale of the Three Factor Eating Questionnaire (TFEQ)-18 (Karlsson, Persson, Sjostrom, & Sullivan, 2000) for the purpose of obtaining a measure of dietary restraint. Participants were then divided into high and low dietary restraint using the scale mid-point (<12: low dietary restraint, n = 52; ≥12: high dietary restraint, n = 92).

#### Other measures

Explicit attitudes to fruits and snacks were assessed with agreement to 10 separate statements: five assessing attitudes to ‘fruit’ (“For me, eating fruit is healthy.”; “For me, eating fruit is bad.”; “For me, eating fruit is enjoyable.”; “For me, eating fruit is pleasant.”; “For me, eating fruit is good.”) and five assessing attitudes to ‘cake or biscuits’. Responses could be marked along 7-point differential scales (anchored from “not at all” to 7 “very”) and reverse scored where appropriate (Hollands, Prestwich, & Marteau, 2011).

### Procedure

Eligible panel members (see ‘Participants’ section) were invited to participate by letter and those interested were asked to log on to the study registration website, give informed consent and complete a short questionnaire which asked for their age, gender, weight, height, and dieting status.

Participants were randomly allocated to one of four groups in a two (prime or no prime) by two (cognitive load or no cognitive load) between-subjects factorial design, and sent an email directing them to the appropriate task website. The task website advised participants that they were being asked to complete a number of unrelated tasks, and asked them to indicate their hunger and thirst levels,. They then completed the tasks in the following order: advert rating task (prime group only); food shopping questionnaire; size estimation task; food preferences task; and end of study questionnaire (including dietary restraint, and explicit attitudes to food).

### Analyses

Size estimates were analyzed using a linear regression model. The model was referenced to the apple, and included dummy variables for the blueberry muffin and book size estimates. The model included main effects for prime, hunger, cognitive load and dietary restraint, and interaction terms between prime and hunger (Hypothesis 1) and prime and cognitive load (Hypothesis 2), as well as prime and restraint.

Fruit choice was analyzed using a logistic regression model with a binomial distribution (the sum of seven binary outcomes). The model included main effects for the prime, hunger, restraint and cognitive load, and interaction terms between prime and hunger (Hypothesis 1), and prime and cognitive load (Hypothesis 2), as well as prime and restraint.

## Results

163 participants were randomized to a study group and 143 completed the testing. Randomization was successful, generating no baseline differences (*p* > 0.1) between groups in BMI, gender, age, dieting status or hunger. Participants chose on average 4.70 (SD 1.78) fruits out of seven in the food preference task.

Hunger ratings were not normally distributed, with 37% of participants giving a rating of 1, indicating they were not hungry. To enable a meaningful analysis, participants were grouped into two hunger levels: scores of 1 and 2 were grouped as ‘no hunger’ (n = 85) and scores of 3 to 7 were grouped as ‘some hunger’ (n = 58).

### Hypothesis 1

Linear regression of the size estimate with reference to the apple revealed no significant effect of prime on size estimate for the apple (Effect (95% CI) = −0.350 cm (−2.76–2.06), *p* = 0.776) and no significant interaction between prime and hunger (Effect (95% CI) = −0.852 cm (−2.99–1.28), *p* = 0.435).

**Fig. 1:**
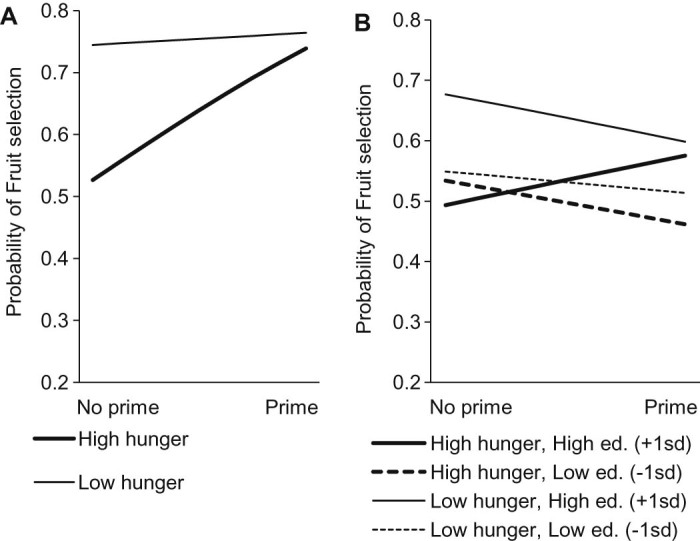
Simple slope analysis illustrating the effects from the logistic regression models of the interaction between prime and hunger in Study 1 (A), and the three-way interactions between prime, hunger and education in Study 2 (B) (Simple slope analysis following Jeremy F. Dawson, 2013). For the current sample, one standard deviation below the mean in terms of education equates with up to 1 A-level, and one standard deviation above the mean with a first degree.

However, consistent with Hypothesis 1, there was a significant interaction between hunger and prime on the number of fruits chosen in the food preference task. There was an overall effect of hunger, in that hunger reduced the odds of fruits being chosen (OR (95% CI) = 0.38 (0.25–0.56), *p* *<* 0.0001). Within the group of participants reporting some hunger, those in the prime condition were more likely to select fruits than those in the non-prime condition (OR (95% CI) = 2.29 (1.33–3.96), *p* = 0.003). The prime had no effect on participants who reported no hunger (OR (95% CI) = 1.11 (0.60–2.04), *p* = 0.736) (Table 2, Fig. 1).

This analysis was repeated controlling for thirst, with thirst treated in the same manner as hunger (‘no thirst’: score 1–2, n = 57; ‘some thirst’: score 3–7, n = 86). No effect of thirst was found on number of fruits chosen (OR (95% CI) = 1.08 (0.80–1.46), *p* *=* 0.619) and controlling for thirst did not alter any of the findings reported above.

### Hypothesis 2

There was no support for Hypothesis 2. Linear regression of the size estimate with reference to the apple revealed no significant interaction between prime and cognitive load (Effect (95% CI) = 0.258 cm (−1.83–2.35), *p* = 0.809) on the size estimate for the apple. Logistic regression of the number of fruits chosen also revealed no interaction between prime and cognitive load (Table 2).

## Discussion

These results provided partial support for Hypothesis 1 – that the prime activated the goal of eating fruits in participants with some hunger. The data suggested that participants with some hunger were less likely to choose fruit, but those who were primed were more likely to choose fruit than those who were not primed. No effects were found on the size estimation task. The results give no support to Hypothesis 2: the prime did not seem to activate a goal of eating fruits and vegetables in participants under cognitive load, either as measured with food preference or with size estimates.

Consistent with the current findings, other studies have also found that hunger reduces the likelihood of purchasing low calorie foods such as fruits or vegetables (Tal & Wansink, 2013) and reduces the likelihood of selecting vegetables over starches or proteins when eating a meal (Wansink, Tal, & Shimizu, 2012). It has been shown that hunger reduces the relative importance of taste preference and increases the relative importance of function attributes, such as portion size or waiting time, in a discrete food choice task (Hoefling & Strack, 2010). So it may be that the prime mitigates the effect of hunger on food choice by either boosting the perception of fruit as more functionally valuable (Veltkamp, Aarts, & Custers, 2008a), or by boosting the relative importance of taste preference in hungry food choice, thus making hungry and non-hungry food choices more similar.

The hypothesis that the prime would increase fruit selection when under cognitive load was not supported. There are two possible explanations. First, it is possible that the cognitive load manipulation was not demanding enough, though previous work used the same task with success (Van Boven & Robinson, 2012). Alternatively, the cognitive load manipulation may have limited impact on food choices when images of food are used, because viewing images of tempting foods elicits fewer affective reactions than would viewing actual food, and it is these reactions which drive selection under cognitive load (Shiv & Fedorikhin, 1999).

The current data elicited a priming effect not contingent on restraint status. Based on these data, it seems possible that a prime that pairs healthy food with a motivational value not specifically related to health could alter food selection in a broader range of participants than hitherto observed in priming studies.

A strength of the current study design was that it tested the use of an intervention – adverts and banners for healthy foods – that is readily transferable to real food shopping environments where advertising material is placed alongside food (though in the case of the adverts, the use of a rating task forced a level of engagement that is greater than would be expected in a real world use of such a prime). The outcome measure, while simplistic, was also similar to certain real world food choices where a limited range of items is available to choose from. The current study was designed to identify a prime with the potential to influence grocery shopping, so while it did not assess priming in a real-world behavior, the findings can inform the design of future studies of real-world food choice.

A limitation of the current study, along with many other demonstrations of priming, was a relatively small sample size. Although the interaction effect size was large, the standard error was also relatively large. This suggests that replication with a larger sample is warranted. Moreover, participants were recruited from a local volunteer research panel, and other studies conducted with the same panel have shown that participants are older, more educated, and leaner than the general population (Forwood, Ahern, Marteau, & Jebb, 2015). The results in the current study might not reflect the behavior of individuals who would benefit most from healthy eating interventions, such as individuals in lower socio-economic groups.

Another limitation of the current study was that the majority of participants were not hungry when they completed the task. Given that hunger was an important predictor of fruit choice and interacted with the effect of the prime, Study 2 used a recruitment strategy that aimed to recruit participants with a wider range of hunger.

## Study 2

Study 2 sought to replicate and explain the findings in Study 1, and to assess whether the findings from Study 1 generalize to a sample more representative of the general population in terms of socio-economic status, age and BMI.

To replicate the observed interaction between prime and hunger, we purposefully sampled participants who had either eaten within the last hour or who had eaten more than two hours prior to study participation. In addition we powered the study to assess hunger as an effect modifier of the prime on fruit choice. Finally, Study 2 sought to extend the findings from Study 1 by exploring hypothesized psychological mechanisms for the effect of the prime.

Two explanations for the observed interactions in Study 1 were considered. First, the prime may be creating a more positive implicit association between health and taste (Raghunathan, Naylor, & Hoyer, 2006; Werle, Ardito, & Trendel, 2011), since the prime pairs healthy food with positive mood and eating tasty foods has been shown to elicit positive moods (Desmet & Schifferstein, 2008; but see also Macdiarmid & Hetherington, 1995). This can be assessed in two ways. First, with an explicit measure of the belief that foods can either be healthy or tasty (Raghunathan et al., 2006; Werle et al., 2011), and second, with an evaluative priming task (Fazio, Sanbonmatsu, Powell, & Kardes, 1986) that uses reaction times to identify the congruency of concepts at an implicit level.

Secondly, the prime may be activating a pre-existing healthy eating goal. Food-related goals influence food choices (Glanz, Basil, Maibach, Goldberg, & Snyder, 1998), and as long as multiple goals are held simultaneously, the prime has the potential to activate one goal over other competing alternatives (Forster et al., 2007).

We tested three hypotheses:

(1) The prime activates the goal of eating fruits and vegetables, as measured by an increased frequency of fruit selection in a food preference task, in participants with high hunger (as per Study 1).
(2) The interaction in Hypothesis 1 is mediated by (a) implicit associations between tastiness and fruit and (b) an explicit belief that healthy foods are not tasty.
(3) The interaction in Hypothesis 1 is moderated by explicit health goals: the interaction is strongest in those with a stronger health goal in relation to food.

## Method

### Design

Study 2 manipulated prime exposure in a between subjects design. The prime factor comprised a prime condition (with adverts and healthy eating banner) and a control (no adverts and non-food banner). In addition to the primary outcomes used in Study 1, all participants in Study 2 also completed measures of explicit food goals, implicit association between healthy foods and taste, and explicit associations between healthy foods and taste.

### Participants

To detect the interaction between prime and hunger on fruit selection seen in Study 1 (OR = 2.29) required a total sample size of 189 participants (calculated using coefficients from Table 2, alpha of 0.05 and power of 0.80 for a logistic regression with a binomial distribution, using G*Power 3.01). Given the interaction effect seen in Study 1 had a large standard error associated with it, to detect a more conservative interaction effect size of one standard error smaller than that seen in Study 1 (OR = 1.53) required a total sample size of 703 participants (calculated in the same way).

Seven-hundred and sixty-four (N = 764) participants were recruited via OnePoll (www.OnePoll.com), an online market research agency (mean (SD) age: 38.5 (14.1) years; BMI: 26.7 (6.2); 64% female; 48% university educated; see Supplementary Table S1). Panel members who were both UK residents and 18 years or older were invited to take part through the agency website. The research agency recruited participants to generate a mix of levels of educational qualification, age, BMI and gender. Participants were compensated for taking part with credits issued within the research agency website. This research was approved by the University of Cambridge Psychology Research Ethics Committee (PRE 2011-57).

### Intervention

As in Study 1, participants in the prime condition were asked to evaluate a series of mock adverts, and were presented with a banner featuring a wide selection of fruits and vegetables when undertaking the food shopping questionnaire, whereas participants in the no prime condition did not complete an advert rating task and were presented with a banner featuring an abstract splash of colored paint. The materials used for the prime intervention were identical to those used in Study 1.

### Measures

Hunger, thirst, size estimation and food preference were measured in the same manner as for Study 1. Size estimates results were not reported due to absence of effect in Study 1.

#### Explicit food goals

These were assessed by responses to two questions: “How important are each of the following for you when choosing what food to eat?” for the goals of ‘health’, ‘cost’, ‘taste’, ‘weight control’ and ‘convenience’ (5-point scale anchored at 1 *very unimportant* to 5 *very important*), and “Which is the most important for you when choosing what food to eat?” (one selection allowed from the same five goals listed above).

#### Implicit association between healthy foods and taste

The evaluative priming task (EPT; Fazio et al., 1986) was used to measure the extent to which participants considered fruits and snacks as compatible with healthy words and with taste words. Each trial consisted of a 500 milliseconds (ms) fixation cross, followed by a word (prime) for 200 ms, then a food image. When the food image appeared, category labels appeared in the top left and right of the screen and participants were asked to respond with a key press (“A” or “L” on the computer keyboard) to sort the food into the appropriate category. The side of the screen used for presentation of each label was randomized between participants.

The food images used were the same 14 foods used in the food preference task; seven fruits and seven snacks. The two category labels used were “fruit” and “pastry/dairy” snack. The prime words used were “natural”, “health”, “wholesome”, “nutritious”, “well-being” as the healthy terms and “yummy”, “tasty”, “appetizing”, “delicious”, “scrumptious” as the tasty terms. The measure of interest in this task was reaction times (the time taken to sort the food images into the two categories).

Mean reaction time (RT) data were collected for four trial types per participant: ‘healthy-fruit’, ‘tasty-fruit’, ‘healthy-snack’, ‘tasty-snack’. Data from error trials and trials with a very short (<200 ms) or very long (>2000 ms) reaction times were excluded from the analysis.

#### Explicit association between healthy foods and taste

Participants completed a two-item scale indicating their belief that healthy food could not be tasty: “Food that is unhealthy generally tastes better.” and “There is no way to make food healthier without sacrificing taste.” (responses on a 5-point scale anchored at 1 *Strongly disagree* to 5 *Strongly agree*) (Raghunathan et al., 2006).

#### Demographic measure

Participants indicated their highest educational qualification: no qualifications, up to four OLevels/GCSE's (aged 16 UK school qualifications) or equivalent, five or more GCSE's/OLevels, up to one A-level (aged 18 UK school qualifications), two or more A-levels, a Bachelor's degree (or equivalent), a post-graduate degree (or equivalent).

### Procedure

Eligible panel members (see ‘Participants’ section) invited to take part in the study clicked on a link within the market research company website to reach the study. At the study website, participants gave informed consent and were screened with a question on recency of eating: “How long ago did you last eat a main meal (breakfast, lunch or dinner)?” with 5 response options (over 3 hours, 2–3 hours, 1–2 hours, 0–1 hours, currently eating)? Only participants who had eaten ≤1 hours ago or ≥2 hours ago were allowed to continue with the study.

Participants completed a short questionnaire which included measures of explicit food goals, self-reported age, gender, weight, height, and dieting status, and indicated their current hunger and thirst levels. Participants were then randomly allocated by the study website (hosted by Qualtrics.com) to one of two groups: prime or no prime, and taken through the tasks in the following order: advert rating (prime group only); food shopping questionnaire; size estimation task; food preferences task; evaluative priming task; and end of study questionnaire.

The evaluative priming task and the food preference task were both completed within a plug-in designed to capture reaction time data over the web (Inquisit 4.0 Web, by Millisecond.com) (De Clercq, Crombez, Buysse, & Roeyers, 2003), and the order of these two tasks was randomized by the program, such that half the participants replicated the order of testing used in Study 1.

### Analyses

The same data analysis methods from Study 1 were used to explore Hypothesis 1. Assuming Hypothesis 1 was confirmed, a causal mediation analysis (Tingley, Yamamoto, Hirose, Keele, & Imai, 2013) was planned to explore whether either the strength of the implicit association between tastiness and fruit, or explicit beliefs that healthy foods are not tasty, mediated the interaction between prime and hunger on fruit selection (Hypothesis 2). A final model was planned to explore whether the interaction in Hypothesis 1 was moderated by explicit health goals (Hypothesis 3). Fruit choice was analyzed using a logistic regression model including main effects for the prime, hunger and health goal rating, and all interaction terms up to the three-way interaction prime and hunger healthy goal rating.

## Results

1288 participants were randomized to a study group and 764 participants completed the testing process. Randomization was successful in terms of equal allocation of recent meal and distant meal participants to each of the four conditions, as well as in terms of gender, age, BMI status, educational qualification and dieting status (all *p* > 0.1). Participants chose on average 3.84 (SD 1.88) fruits out of seven in the food preference task.

Hunger was operationalized by self-reported hunger as in Study 1: no hunger (scoring 1–2, n = 418) and some hunger (scoring 3–7, n = 346). As for study 1, hunger ratings were not normally distributed, with 39% of participants giving a rating of 1, indicating they were not hungry. There was a significant main effect of the order of testing (evaluative priming task before or after food preference task) on the odds of selecting fruits in the food preference task (OR (95% CI) = 1.171 (1.05–1.30), *p* = 0.004). We therefore controlled for order of task completion in testing Hypothesis 1.

### Hypothesis 1

Hypothesis 1 was not supported: there was no significant interaction between hunger and prime on the number of fruits chosen in the food preference task (Table 2). Repeating the logistic regression model without the non-significant interaction terms revealed an overall effect of hunger such that some hunger resulted in fewer fruits being chosen, which replicated the finding from Study 1.

Hypotheses 2 and 3, which were predicated on Hypothesis 1 being supported, were not tested. We therefore conducted further analyses to explore why the results from Study 1 were not observed in Study 2.

### Comparing study 1 and 2

Studies 1 and 2 were similar in terms of the measures used but differed in some aspects of design and the type and number of participants. The design difference (order) was controlled for in the analysis for Study 2, so it was unlikely to explain the difference in effects. The smaller number of participants used in Study 1 may mean that the findings from Study 1 represent a false positive, i.e. a Type 1 error. However, the most notable and planned difference between Study 1 and Study 2 was the participants, suggesting that the effects seen in Study 1 may not generalize to a wider population.

Study 2 explicitly sought to recruit participants from a demographic background more representative of the general population than those in Study 1. Comparing the study samples revealed significant differences in BMI, age, and diet status, but not gender (Supplementary Table S1). Study 1 participants were leaner, older and less likely to be on a diet to control their weight than Study 2 participants. While data on education were not collected as part of Study 1, it is likely that Study 1 participants were also more educated than Study 2 participants. In another study, sampling from the same panel as Study 1 (Forwood et al., 2015), 74% of participants (n = 155) completed their education after the age of 19, or were still in education and aged 19 or more. By comparison, only 48% of participants in Study 2 reported having a qualification gained after leaving school (degree or equivalent, or higher).

### Demographic interactions with prime and hunger

Given the clear differences in participant demographic characteristics between Studies 1 and 2 (Supplementary Table S1) in terms of age, education and BMI, analyses were conducted to assess whether these demographic differences interacted with the experimental intervention – prime and hunger – to influence the endpoint of this study – number of fruits chosen in the food preference task. This analysis tests the hypothesis that the effect of the prime seen in Study 1 was specific to participants with the demographic characteristics recruited for Study 1 (older or more educated or leaner individuals), and failure to see these effects in Study 2 was the result of the effects seen in Study 1 not generalizing to participants with broader demographic characteristics.

A logistic regression model of fruits chosen with three-way interactions of prime and hunger with education level, age and BMI revealed significant three-way interactions for prime and hunger with education (OR (95% CI) = 1.42 (1.13–1.79), *p* = 0.003), but not for age (OR (95% CI) = 1.246 (0.98–1.58), *p* = 0.068) or BMI (OR (95% CI) = 0.860 (0.68–1.09), *p* = 0.219). Plotting the simple slope analysis using the coefficients from the logistic regression model (J.F. Dawson, 2013) illustrates the interaction effect (Fig. 1B): individuals who were both hungry and more educated showed increased fruit selection in response to the prime (OR (95% CI) = 1.42 (1.13–1.79), *p* = 0.003), whereas all other individuals showed no change in fruit selection in response to the prime. Odds ratio (95% CI) of the effect of prime on fruit selection are as follows: for no hunger and lower education participants: 0.889 (0.79–1.14), *p* = 0.560; for hungry and lower education participants: 0.906 (0.72–1.13), *p* = 0.387; and for no hunger and higher education participants: 0.951 (0.82–1.11), *p* = 0.519.

### Predictors of fruit choice

In order to assess the predictors of fruit choice, an elastic net regression model (Supplementary Table S2) was chosen by cross validation from a full model of fruit choice by prime, hunger, order, gender, BMI, age, education, all food goal measures, all evaluative priming task trial type reaction times and the rating of the belief of an association between health and taste. This model is designed to handle large numbers of variables and select the more influential variables, even if some of the variables are inter-correlated. The model identified a number of factors that predicted fruit choice: e.g. fruit was less likely to be chosen by younger or less educated individuals, those less likely to select health as their primary goal, those who believed that healthy foods cannot be tasty, those giving lower ratings to a weight control goal or those giving higher ratings to a convenience goal.

## Discussion

The interaction between prime and hunger observed in Study 1 was not observed in Study 2 (Hypothesis 1): there was no evidence that the prime activated the goal of eating fruits and vegetables in a nationally representative sample of participants, as measured by an increased frequency of fruit selection in a food preference task in hungry participants. As in Study 1, a main effect of hunger was found on the frequency of fruit selection, with hungrier participants being less likely to select fruit.

Given the absence of support for Hypothesis 1, it was not possible to conduct the planned analyses into the mechanism for the effect of prime to test Hypotheses 2 and 3.

Comparison of the design, measures and participants used in Studies 1 and 2 revealed some key differences. As intended, Study 2 included a broader range of participant ages, BMI status and socio-economic status (as indicated by highest educational qualification), and further analysis revealed that the prime increased preference for fruit only in hungry and more educated participants, as seen in Study 1. In contrast, less educated participants showed no change in fruit selection in response to the prime.

Study 2 can therefore be viewed as a replication and a refinement of Study 1: it showed that the effects seen in Study 1 were likely specific to the demographic group recruited for Study 1, i.e. those with a higher level of education, and were not generalizable to the whole population. The primes used in the current research are therefore poor candidates for a population-wide health intervention because they would likely benefit (through increased fruit selection) one sector of the population only, without helping other members of the population. This is compounded by the observation that those who show no benefit from priming – less educated individuals – already show less fruit and vegetable intake than those who would benefit, i.e. more educated individuals (current study; Pechey et al., 2013; Shohaimi et al., 2004). Adopting primes such as those explored in the current study as a population-wide intervention could therefore widen a pre-existing social inequality in fruit and vegetable intake. Furthermore, given the known associations between fruit and vegetable intake and the incidence of non-communicable diseases such as diabetes (Cooper et al., 2012) and all-cause mortality (Khaw et al., 2001), the use of such a prime could exacerbate existing social inequalities in life expectancy (Graves, 2011).

Independently of the effect of the prime, the current study highlighted a number of demographic predictors of fruit choice in that older and higher-educated participants were more likely to select fruit than snacks. These observations are consistent with previous data showing differences in food purchasing and consumption by SES (Pechey et al., 2013; Shohaimi et al., 2004) and age (Glanz et al., 1998; Logue & Smith, 1986). Importantly, these effects were observed while controlling for a number of psychological constructs, including measures of food beliefs and goals that also predicted fruit choice. This suggests that age and education appear to have an impact on food preferences not explained by the psychological variables that predicted fruit selection in the current study, including explicit food goals and the belief that unhealthy foods generally taste better (Raghunathan et al., 2006), in line with previous studies (Forwood, Walker, Hollands, & Marteau, 2013; Glanz et al., 1998).

Although the current study captured a range of psychological mediators or moderators with the aim of exploring the psychological mechanism of the prime, the failure to find the anticipated pattern of effects made this analysis unviable. The finding of the SES moderation of the effect of the prime was not anticipated and although of great interest, the study was not designed or powered to cast light on the psychological basis for SES moderation of the prime so further hypothesis-based research designed specifically for this purpose is required.

## General discussion

This study provides evidence that a prime which promotes fruit and vegetable consumption, without appealing to healthiness, could activate a goal of healthier eating independently of restraint status, as measured by food preference. No similar effects were found with size estimates as a measure of goal activation. Both studies reported here show this effect of the prime only in more educated participants with some hunger. The effects did not generalize to other participants – either less hungry or less educated individuals. Subject to replication under real-world conditions, these data suggest that a prime that pairs healthy food with positive mood or social norms and without reference to healthiness could have a positive effect on food choices in more educated members of the population. This could, however, have the adverse consequence of increasing inequalities in health outcomes within the population, given that the prime only increased fruit selection in these individuals who already show higher than average rates of fruit selection (current data, Pechey et al., 2013; Shohaimi et al., 2004).

The observation that priming in higher educated individuals – an individual-level measure of SES – is contingent on their biological motivational state is in keeping with the current literature on priming a goal (Veltkamp et al., 2008a). In this literature, priming is described as rendering more accessible a deprivation-reducing behavior (Veltkamp et al., 2008a), and thereby facilitates its action when individuals are in a state of deprivation. Independently of priming, hunger has also been shown to increase the extent to which foods are chosen based on their functional attributes and reduce the extent to which they are chosen based on an idiosyncratic taste preference (Hoefling & Strack, 2010) explaining the increased selection of higher energy foods over lower energy alternatives under deprivation (Tal & Wansink, 2013; Wansink et al., 2012). In the current study, therefore, exposure to primes may mitigate the effect of hunger on food choice by rendering more accessible the deprivation-reducing capacity of eating fruit.

Understanding the differential impact of the prime as a function of education is more challenging. The above explanation of the effect of the prime suggests that the prime is rendering more accessible the deprivation-reducing capacity of eating fruit, implying some pre-existing knowledge of the deprivation-reducing capacity of eating fruit for the prime to act on. One possible explanation of the observed social differences is therefore that individuals with different education levels have different beliefs of the expected satiety offered by fruit. Studies into expected satiety have shown that the experience of eating a food to satiety increases the expected satiety for that food (Irvine, Brunstrom, Gee, & Rogers, 2013), and given lower SES individuals purchase less fruit (Pechey et al., 2013), they are also less likely to consume fruit to satiety, though further research is needed to test these ideas.

The priming literature offers few examples of studies that have explored education or other demographic moderators of the effects of primes (Cesario, 2014). One potential source of information on this issue is real food advertising. We conducted a small-scale analysis of the wording and imagery used in 94 food advertisements placed in UK magazines in December 2013 after the current studies were completed. This showed significant differences in the wording used in adverts contained in magazines targeting lower SES populations compared to those targeting higher SES populations (as determined by national readership figures, www.nrs.co.uk). From the adverts studied, terms indicating quality (e.g. ‘great, ‘best’) or cost (e.g. ‘3 for £3’) appeared more often in adverts in magazines targeting lower SES populations, whereas terms indicating authenticity (e.g. ‘real Italian’) or pleasure (e.g. ‘a moment of bliss’) appeared more often in adverts in magazines targeting higher SES populations. No similar differences were seen in the imagery used, with the predominant image being the product. Discussions with advertisers suggest that adverts are designed to target barriers to food consumption, and are known to be more effective for individuals with prior experience of the product (Walker: personal communication). Both barriers to food and prior consumption of food will likely differ for individuals from different demographic groups, and interestingly echo findings from the priming literature where it has been shown that an individual's identity and level of chronic goal activation both determine the extent to which one assimilates or contrasts with a prime (Hart & Albarracin, 2009; Ledgerwood & Chaiken, 2007).

The absence of an effect of hunger on food preference for less educated lower SES individuals was not expected given previous studies (Tal & Wansink, 2013; Wansink et al., 2012). On the basis that hunger alters the food attributes that drive choice (Hoefling & Strack, 2010), these data may suggest that more and less educated individuals differ in their relative ranking of foods on the attributes that drive choice: for instance in terms of taste preference hunger would reduce fruit selection if fruit is liked more than snacks, but not if fruit is liked to the same extent as snacks. Alternatively the relative importance of different attributes in determining food choice may differ between education groups. In terms of taste preference, some preliminary data support this suggestion showing higher liking for fruit than cake in higher SES individuals but not in lower SES individuals (Pechey, Monsivais, Ng, & Marteau, 2015). In terms of attributes that determine food choice, more research is needed to explore these ideas further. Given the interplay between hunger and priming that has already been discussed, a better understanding of the impact of hunger on food choice may provide a more robust foundation for understanding priming as well.

The studies conducted here have a number of design strengths and limitations. Both Study 1 and Study 2 employed a between participant randomized design, and Study 2 was conducted within a large and demographically varied sample, and is therefore able to inform discussion of the use of primes at a population level. Both studies also used an intervention and an outcome measure that were comparable with real world food choice environments.

The studies also had limitations relating to their design. First, the intervention comprised multiple components: three advertisements and a banner. We do not know which of these singly or together explained the impact of the prime. Second, the studies did not establish whether participants were aware of a link between exposure to the prime and their subsequent food choice behavior – and it is therefore not possible to say to what extent the reported effects result from the prime automatically influencing behavior, and to what extent they result from demand characteristics that may be greater in some participants. In addition, the studies did not establish whether the prime activated a goal (evidence of a goal priming mechanism) or merely enhanced the accessibility of semantic constructs related to this goal (semantic priming) (Forster et al., 2007) for instance, by exploring the effect of goal fulfillment on goal activation (Forster et al., 2007). These questions were beyond the scope of the current research which sought to capture whether effects within a convenience sample generalize to a nationally representative sample of participants. A final limitation of the current study design is that the outcome measure reflects expressed preference rather than actual food choice. Participants would have been aware that they were not going to eat the foods selected, given that these experiments were conducted online, and replication of the current findings in a real food choice study is a necessary next step in this research. There is evidence that food preference measures such as this correlate with actual food intake (Drewnowski & Hann, 1999).

Recent debate within the social and goal priming literatures has raised doubts over whether reported effects can be replicated (Bower, 2012; Doyen, Klein, Pichon, & Cleeremans, 2012; Yong, 2012), fueling a wider discussion over replication within psychological research in general (Pashler & Wagenmakers, 2012). One upshot of this debate is that it has been suggested that failures to replicate social priming effects – a fundamentally social phenomenon – are due to these effects being sensitive to the trait and state of the individual experiencing the prime, and that this has not been described and explored in sufficient depth to fully understand and predict the social contingencies under which priming effects are observed (Cesario, 2014).

The findings of the current research are certainly consistent with priming effects being sensitive to individual trait and state differences: in this case education and hunger. But more importantly they flag up the possibility that applying findings from basic research conducted on a subset of the population (Henrich, Heine, & Norenzayan, 2010) to public health will generate interventions that benefit that subset but may not benefit the rest of the population. So while priming holds promise as a population-wide intervention for improving the healthiness of everyday behaviors, a more robust body of evidence is needed on the trait and state modifiers of the effects of experiencing a prime for this promise to be realized. Without this, there is a danger that in conceiving and using priming interventions across a population, we may unintentionally increase inequalities in the targeted behavior.

## Figures and Tables

**Table 1:** Summary of current studies that evaluate the effect on food choice or intake of primes that are feasible for use within real purchasing environments (excluding subliminal primes).

Study	Location	Prime	Outcome	Participants recruited from (mean age)	Effects in
Boland et al. (2013)	Laboratory	Healthy food TV ad	↓M&Ms eaten	Students (19.3)	All
Buckland et al. (2013)	Laboratory	An orange	↓Snacks eaten on taste test	University campus (26.3)	Restrained only
Coelho et al. (2009)	Laboratory	Cookie odors	↓Cookies eaten	Students (n/a)	Restrained only
Gaillet et al. (2013)	Laboratory	Fruit odors	↑Fruit and vegetable selection	Normal weight (27.5)	All
Gaillet-Torrent et al. (2014)	Laboratory	Fruit odors	↑Fruit dessert selection	Normal weight (26.0)	All
Papies and Hamstra (2010)	Butcher's shop	Diet recipe poster	↓Meat snacks eaten	Customers (56)	Restrained only
Papies et al. (2013)	Supermarket	Diet recipe flier	↓Unhealthy snacks purchased	Lower SES customers (54.2)	Overweight only
van Koningsbruggen et al. (2011)	Laboratory	Healthy magazine covers	↑Healthy eating goal activation	Unreported (28.7)	Restrained only

**Table 2:** Logistic regression models indicating the odds ratio of selecting fruit by randomized group in Study 1 and Study 2.

	Study 1 OR (95% CI)	Study 2, model 1 OR (95% CI)	Study 2, model 2 OR (95% CI)
(Intercept)	2.92 (1.87–4.62)***	1.26 (1.05–1.51)*	1.27 (1.10–1.47)**
Prime (ref no prime)	1.11 (0.60–2.04)	0.95 (0.74–1.20)	0.923 (0.83–1.03)
Restraint (ref low restraint)	1.07 (0.72–1.59)	0.95 (0.80–1.13)	0.98 (0.87–1.11)
Some hunger (ref no hunger)	0.38 (0.25–0.56)***	0.91 (0.78–1.06)	0.845 (0.76–0.95)**
Load (ref no load)	0.86 (0.58–1.25)	n/a	n/a
Order (ref food preference first)	n/a	1.18 (1.06–1.31)**	1.18 (1.05–1.31)**
Prime × restraint	0.72 (0.41–1.27)	1.07 (0.83–1.37)	n/a
Prime × hunger	2.29 (1.33–3.96)**	0.87 (0.70–1.08)	n/a
Prime × load	0.88 (0.51–1.51)	n/a	n/a

Study 2: model 1 contains interaction terms, model 2 contains no interaction terms.

* *p* < 0.05, **p < 0.01, ***p < 0.001.
