# Supplementary material for: Priming healthy eating. You can't prime all the people all of the time
Source: Appetite. 2015 Jun 1;89:93–102. doi: 10.1016/j.appet.2015.01.018 (PMC4544036; doi:10.1016/j.appet.2015.01.018)
Supplement: Appendix S1 — Fig. S1 and Tables S1–S2. [file mmc1.docx]

# Supplementary materials


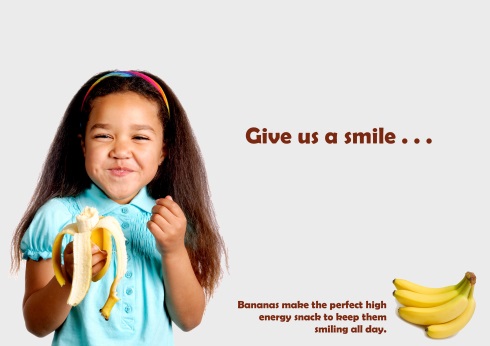

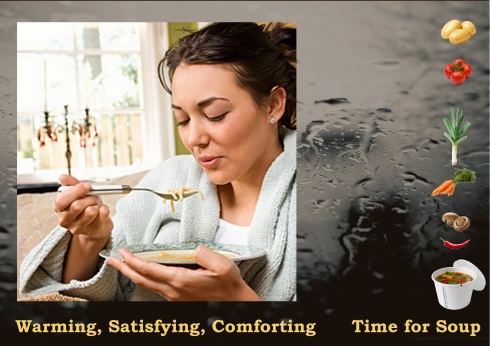


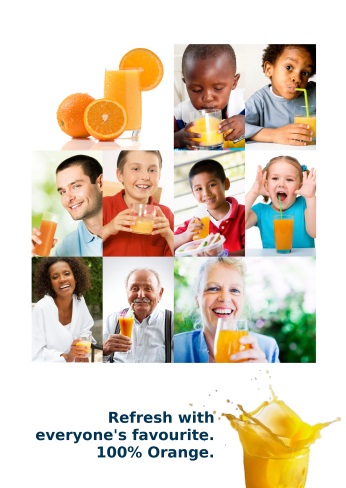


Supplementary Figure 1: Fruit and vegetable advertisements used as primes (created by SEF using GIMP ([www.gimp.org](http://www.gimp.org)) and photographic images from iStockPhotos.com).

Supplementary Table 1: Comparison of participant characteristics between Study 1 (Local participant panel) and Study 2 (National market research agency)

|  | Study 1  (n=143) | Study 2  (n=764) |  |
| --- | --- | --- | --- |
| BMI status [N (%)] |  |  |  |
| Lean (18 to 25) | 89 (62.2) | 345 (45.2) | *** |
| O’weight (25 to 30) | 36 (25.2) | 242 (31.7) |  |
| Obese (>30) | 18 (12.6) | 177 (23.2) |  |
| BMI [mean (sd)] | 25.3 (6.9) | 26.7 (6.2) | * |
| Age [mean (sd)] | 43.6 (14.96) | 38.5 (14.1) | *** |
| Gender: female [N (%)] | 98 (68.5) | 489 (64.0) | n.s. |
| Dieting [N (%)] | 28 (19.6) | 289 (37.8) | *** |
| High dietary restraint [N (%)] | 88 (61.5) | 711 (93.1) | *** |
| Education [N (%)] |  |  | *** |
| Up to 4 GCSE’s | n/a | 99 (13.3) |  |
| Up to 1 A-level | n/a | 120 (15.7) |  |
| 2+ A-levels | n/a | 175 (23.0) |  |
| First Degree | n/a | 244 (31.9) |  |
| Further Degree | n/a | 123 (16.1) |  |
| Hunger Rating [1-7 (sd)] | 2.45 (1.53) | 2.73 (1.81) | n.s. |
| Apple size [mean (sd)] | 7.20 (2.18) | 7.46 (3.11) |  |
| Muffin size [mean (sd)] | 7.75 (2.64) | 7.60 (3.10) |  |
| Book size [mean (sd)] | 10.98 (4.27) | 10.4 (5.78) |  |
| Fruits Chosen [0-7 (sd)] | 4.70 (1.78) | 3.84 (1.88) | *** |

*p<0.05, **p<0.01, ***p<0.001

Supplementary Table 2: Elastic Net Regression of number of fruits chosen. The model presented here is the simplified model chosen based on stepwise selection using AIC from the full model (alpha = 0.5, lambda chosen through cross validation).

|  | OR (95% CI) |
| --- | --- |
| (Intercept) | 3.24 (2.14 – 4.92) *** |
| Prime (ref no prime) | 0.91 (0.81 – 1.02) |
| Gender (ref female) | 1.16 (1.03 – 1.31) * |
| Order (ref Food Preference first) | 1.14 (1.02 – 1.28) * |
| Education (standardized) | 1.13 (1.06 – 1.19) *** |
| Age (standardized) | 1.21 (1.14 – 1.29) *** |
| Restraint (ref low restraint) | 0.90 (0.70 – 1.16) |
| Explicit Belief Healthy ≠ Tasty | 0.86 (0.83 – 0.89) *** |
| Health as primary goal | 1.27 (1.08 – 1.50) ** |
| Taste goal rating | 0.95 (0.88 – 1.02) |
| Health goal rating | 1.06 (0.97 – 1.15) |
| Weight goal rating | 1.09 (1.03 – 1.16) ** |
| Convenience goal rating | 0.91 (0.85 – 0.97) ** |
| Cost goal rating | 0.98 (0.91 – 1.06) |
| Implicit Health-Fruit (standardized) | 0.93 (0.88 – 0.99) * |

. p<0.1, *p<0.05, **p<0.01, ***p<0.001
